# Utility of thromboelastogram in cardiac surgery in Jacobsen syndrome associated with platelet dysfunction: a case report

**DOI:** 10.1186/s40981-022-00557-w

**Published:** 2022-08-22

**Authors:** Chikashi Takeda, Akiko Hirotsu, Gento Yasuhara, Akito Mizuno, Kenichiro Tatsumi, Shuji Kawamoto

**Affiliations:** Department of Anesthesia, Kyoto University Hospital, Kyoto, 606-8507 Japan

**Keywords:** Chromosome 11, Hemostatic capacity, Jacobsen syndrome, Thrombocytopenia, Viscoelasticity

## Abstract

**Background:**

Jacobsen syndrome is a rare genetic disorder with multiple congenital anomalies and platelet abnormalities caused by chromosome 11 deletion.

**Case presentation:**

A 7-month-old boy with thrombocytopenia underwent ventricular septal defect closure. At the beginning of surgery, the platelet count was 168 × 10^3^/μL, and heparinized kaolin with heparinase reaction time (HKH-R), which represents clot formation time, was prolonged at 30.4 min. Platelet transfusion was continued, and at the end of surgery, the platelet count and HKH-R values improved to 215 × 10^3^/μL and 15 min, respectively.

**Conclusions:**

As anesthetic management of patients with abnormal platelet function, the viscoelasticity test might be useful in evaluating hemostatic capacity.

## Background

Jacobsen syndrome (JS) is a group of rare genetic disorders associated with multiple congenital anomalies and mental retardation caused by the partial deletion of the long arm of chromosome 11 [1, 2]. These diseases are associated with hematopoietic disorders, such as thrombocytopenia and platelet dysfunction, which are present at birth [3, 4]. Abnormal bleeding such as cerebral hemorrhage has been reported to occur even when platelet counts are normal [5]. The perioperative use of thromboelastography (TEG), which measures blood viscoelasticity in real time using whole blood, helps in planning for blood product administration [6, 7]. However, no studies have investigated how abnormalities in platelet count and function affect TEG results in patients with JS.

Herein, we describe a case of intraoperative hemostatic control using comprehensive hemostatic assessment focusing on platelet function by TEG® 6s (Haemonetics, Massachusetts, USA) [8] during the ventricular septal defect (VSD) closure in a JS patient who had abnormal platelet function despite a normal platelet count as well as coagulation function tests.

Written consent was obtained from the parent of the patient for publication of this case report.

## Case presentation

A 1-day-old male infant was transferred to our hospital for heart murmur and progressive hypercapnia. He was born via spontaneous vaginal delivery at 36 weeks and 3 days of gestation with a birth weight of 2700 g. He had no remarkable family history, and the pregnancy course was uneventful. He was noted to have a high prominent forehead, low-set ears, a thin upper lip, and retrognathia. There were no abnormalities in the central nervous system, digestive system, and urinary system. On admission, the following thrombocytopenia and coagulation abnormalities were found. Platelet count was 39 × 10^3^/μL, prothrombin time-international normalized ratio (PT-INR) was 1.65, activated partial thromboplastin time (APTT) was 48.8 s, and fibrinogen was 168 mg/dL. Aortic arch reconstruction and ductus arteriosus ligation were performed at 6 days of age. He developed intratracheal hemorrhage on postoperative day 4 and underwent emergency pericardial drainage for pericardial tamponade on postoperative day 11. He also had intraventricular hemorrhage and pancytopenia requiring regular transfusions of platelet concentrates (PCs) and red blood cells (RBCs). A bone marrow test was performed at 2 months of age to investigate the cause of pancytopenia. Genetic testing of bone marrow cells revealed a partial deletion of chromosome 11, leading to the diagnosis of JS. The levels of coagulation factors 8 and 9, von Willebrand, and proteins C and S were all within normal limits. Further close examinations of platelet function and morphology were performed in the fourth month of life after the diagnosis of JS was confirmed. A peripheral blood smear showed large platelets, and mean platelet volume (MPV) was 13 fL (normal: 7–11 fL). Platelet aggregation test showed no aberrant adenosine diphosphate (ADP) aggregation, but collagen aggregation was clearly decreased.

**Fig. 1:**
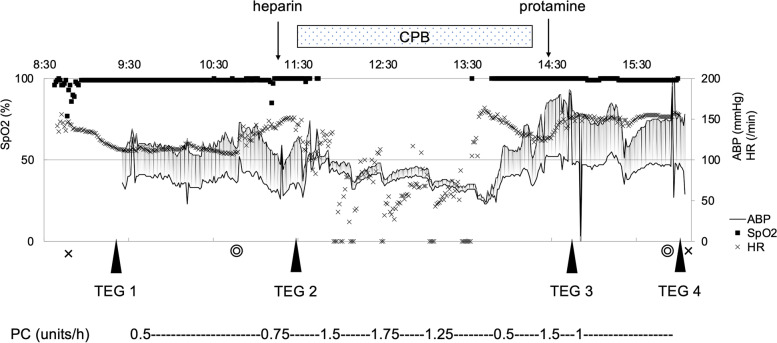
Anesthetic chart during surgical ventricular septal defect closure. Platelet function measured with thromboelastography (TEG® 6s) at the beginning of surgery (TEG 1), before CPB start (TEG 2), at the time CPB withdrawal (TEG 3), and after the end of surgery (TEG 4). We continued transfusion with PC during surgery. One PC unit contains ≥ 0.2 × 10^11^ platelets. *ABP* arterial blood pressure, *CPB* cardiopulmonary bypass, *HR* heart rate, *PC* platelet concentrates

At 7 months of age (height 62.4 cm, weight 4998 g), VSD closure was planned for progressive pulmonary hypertension. Platelet count on readmission was 78 × 10^3^/μL, and five PC units were transfused each of 5 days and 1 day before surgery. Immediately prior to VSD closure, platelet count was normal at 156 × 10^3^/μL, and coagulation function tests were also normal with PT-INR 1.04, APTT 37.6 s, and fibrinogen was 159 mg/dL, slightly below normal. We planned to maintain a platelet count > 100 × 10^3^/μL, also to monitor platelet function using TEG® 6s PlateletMapping® (Fig. 1).

**Fig. 2:**
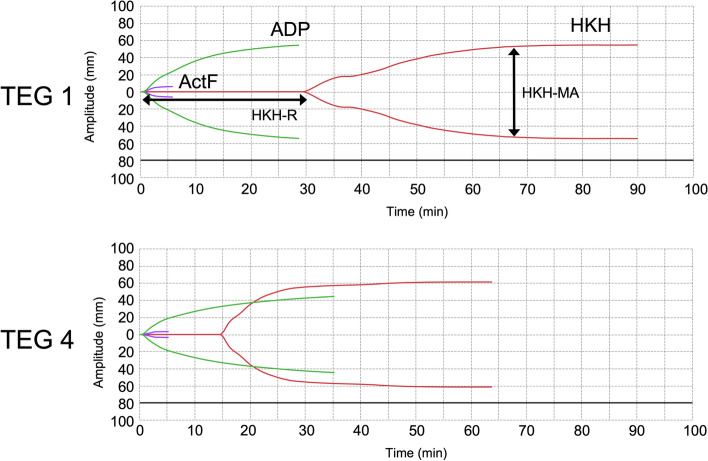
Raw data of thromboelastography (TEG) before (TEG 1) and after surgery (TEG 4). The normal value of HKH-R is 4.2–9.8 min, and the normal value HKH-MA is 53–68 mm. The HKH-R was prolonged preoperatively (TEG 1) and shortened postoperatively (TEG 4) by continued intraoperative platelet transfusions. *HKH* heparinized kaolin with heparinase, *ActF* activator F, *ADP* adenosine diphosphate

Anesthesia was maintained with sevoflurane, fentanyl, and midazolam after uneventful induction with inhalational sevoflurane and tracheal intubation. The platelet count at the beginning of surgery was 168 × 103/μL. Although the platelet function showed a normal clot strength as indicated by the maximal amplitude for heparinized kaolin with heparinase (HKH-MA) of 53.1 mm (normal value: 53–68 mm), clot formation time by HKH reaction time (HKH-R) was prolonged to 30.4 min (normal value: 4.2–9.8 min) (TEG 1; Table 1, Fig. 2). After systemic heparinization, PCs were continuously administered during cardiopulmonary bypass (CPB) at 0.5 U/h. Although platelet count was fewer and HKH-R was longer than the normal range before CPB (TEG 2), both have improved after completion of CPB and surgery (TEGs 3 and 4, respectively). We administered 50 mL of RBCs and 150 mL of fresh frozen plasma (FFP) after CPB until the end of the surgery. Surgery and anesthesia lasted 5 h 8 min and 7 h 32 min, respectively. Intraoperative hemorrhage was approximately 66 mL, and 50 mL of RBCs, 150 mL of FFP, and 150 mL of PCs were transfused. Extubation was performed on postoperative day 1, and the patient was discharged on postoperative day 17. On postoperative day 22, the patient was readmitted to the hospital for subdural hematoma and underwent platelet transfusion.

**Table 1:** Changes in platelet function measured with TEG® 6s during operation

Parameters	Measure point				Range
	TEG 1	TEG 2	TEG 3	TEG 4	
HKH-R (min)	30.4	15	10.8	15	4.2–9.8
HKH-K (s)	9.5	2.6	2.2	2.3	1.0–2.9
HKH-angle (degree)	42.9	60.5	65.5	65.3	57–75
HKH-MA (mm)	53.1	64.5	57.1	57	53–68
ActF-MA (mm)	6.1	9.6	2.3	3.4	2–19
ΔMA (mm)	47	54.9	54.8	53.6	
ADP-MA (mm)	54.4	56.3	31.3	44.6	45–69
ADP inhibition (%)	0	14.9	47.1	23.1	0–17
PLT (× 10^3^/μL)	168	112	133	215	-
Hb (g/dL)	8.1	8.2	10.2	10.2	-

TEG1, TEG2, TEG3, and TEG4 represent preoperative, pre-cardiopulmonary, post-cardiopulmonary, and postoperative measurements, respectively. *Abbreviations*: *TEG* Thromboelastography, *HKH* Heparinized kaolin with heparinase, *R* Reaction time, *K* Coagulation time, *angle* α angle, *ActF* Activator F, *MA* Maximum amplitude, *ADP* Adenosine diphosphate, *PLT* Platelet, *Hb* Hemoglobin

## Discussion

JS is a genetic disorder characterized by facial dysmorphology, cardiac disease, and thrombocytopenia. Its incidence is approximately 1 per 100,000 live births [9, 10]. Diagnosis is frequently based on clinical features and confirmed by karyotyping. Cardiac malformations are present in more than 50% of the patients diagnosed with JS, with two-thirds having VSDs or mitral or aortic valve abnormalities [11]. In addition, platelet abnormalities are frequently observed in JS. The Paris-Trousseau platelet syndrome is present in 95% of patients with JS [3, 4]. Platelet abnormalities manifest as thrombocytopenia or pancytopenia during infancy, but our patient presented with thrombocytopenia from birth and continued to have bleeding events and pancytopenia after surgery leading to the diagnosis of JS. Brain hemorrhages are frequently accompanied in patients with JS as observed in our patient [5].

The TEG test uses whole blood and has been widely utilized in evaluating coagulation function within 10–15 min in the operating room [12]. Intraoperative TEG may help reduce perioperative blood loss, avoid blood transfusions, and determine the strategy of blood product and hemostatic agent administration [7, 13]. In TEG® 6s, the Global hemostasis® cartridge performs conventional coagulation tests, and the PlateletMapping® cartridge measures platelet function [8]. In the current case, we used PlateletMapping® to evaluate platelet function. The HKH in PlateletMapping® is the same as the CKH (citrated kaolin with heparinase) in Global hemostasis®. It contains heparinase that neutralizes heparin and measures thrombin formed by kaolin and clot strength formed by fibrin.

Platelet count was normalized preoperatively and managed intraoperatively to keep platelet count above 100 × 10^3^/μL with reference to platelet count and TEG® 6s. Before surgery, we detected the normal HKH-MA but prolonged HKH-R in our JS patient. This implies a normal maximum hemostatic capacity but a longer time to stop bleeding. This is consistent with the fact that JS is considered a granular release disorder because of the presence of giant granules in the smear [2, 4]. In general, HKH-R prolongation suggests a deficiency of coagulation factors. However, the maximal amplitude evaluated by activator F (ActF-MA), which reflects fibrinogen concentration, was within the normal range, and there were no coagulation factor abnormalities in the preoperative examination. Therefore, the prolonged HKH-R in this case may reflect abnormal platelet function such that only collagen aggregation is reduced while ADP aggregation is preserved.

Platelet transfusion was continued. HKH-R before the start and at the end of CPB improved to 15 min and 10.8 min, respectively. With this, we could perform anesthesia management and confirm that platelet transfusion improved the rate of platelet clot generation. Additionally, ActF-MA remained normal throughout the surgery. HKH-MA–ActF-MA, which indicates the clotting ability of platelets alone, also tended to improve throughout the surgery, suggesting the importance of platelet transfusion in this case.

The percent ADP inhibition increased to 47.1% after CPB withdrawal and improved thereafter (Table 1). Platelet count and ADP aggregation capacity have been reported to decrease after CPB to 57% and 10%, respectively [14]. The CPB time in our patient was approximately 2 h, but the increase in ADP inhibition may be due to CPB.

In conclusion, we presented a case of VSD closure in a JS patient with platelet abnormalities. As platelet count and platelet function need to be carefully monitored during the anesthetic management of patients with JS, a viscoelasticity test using TEG® 6s is a good tool to evaluate hemostatic capacity.

## Data Availability

The relevant data for this case report are unavailable for public access because of patient privacy concerns.

## Abbreviations

ActF: Activator F
ActF-MA: Maximal amplitude evaluated by activator F
ADP: Adenosine diphosphate
APTT: Activated partial thromboplastin time
CKH: Citrated kaolin with heparinase
CPB: Cardiopulmonary bypass
FFP: Fresh frozen plasma
JS: Jacobsen syndrome
HKH: Heparinized kaolin with heparinase
HKH-MA: Maximal amplitude for heparinized kaolin with heparinase
HKH-R: Reaction time for heparinized kaolin with heparinase
MPV: Mean platelet volume
PC: Platelet concentrate
PT: Paris-Trousseau platelet syndrome
PT-INR: Prothrombin time-international normalized ratio
RBCs: Red blood cells
TEG: Thromboelastography
VSD: Ventricular septal defect
